# Mitigation of radiation-induced gastro-intestinal injury by the polyphenolic acetate 7, 8-diacetoxy-4-methylthiocoumarin in mice

**DOI:** 10.1038/s41598-019-50785-x

**Published:** 2019-10-01

**Authors:** Kavya Venkateswaran, Anju Shrivastava, Paban K. Agrawala, Ashok K. Prasad, Sagolsem Chandrika Devi, Kailash Manda, Virinder S. Parmar, Bilikere S. Dwarakanath

**Affiliations:** 1 Division of Metabolic Cell Signalling Research, Institute of Nuclear Medicine and Allied Sciences, Brig. S. K. Mazumdar Marg, Lucknow Road, Delhi, 110054 India; 2 Department of Zoology, University of Delhi, Delhi, 110007 India; 3 Bioorganic Laboratory, Department of Chemistry, University of Delhi, Delhi, 110007 India; 4 Department of Chemistry and Environmental Science, Medgar Evers College, The City University of New York, New York, USA; 5 Present Address: Department of R&D, Shanghai Proton and Heavy Ion Center, Shanghai, 201321 China

**Keywords:** Phenotypic screening, Natural products

## Abstract

Radiation-induced intestinal injury (RIII) constitutes a crucial clinical element of acute radiation syndrome with life-threatening implications posing challenges in devising effective medical countermeasures. Herein, we report the potential of 7, 8-diacetoxy-4-methylthiocoumarin (DAMTC) to mitigate RIII following total-body irradiation (TBI) in C57BL/6 mice and underlying mechanisms. Administration of DAMTC 24 hours post TBI facilitated structural reconstitution and restoration of functional absorption linked to alleviation of radiation-induced apoptotic death of intestinal crypt progenitor/stem (ICPS) and villus stromal cells through induction of Bcl-2 family-mediated anti-apoptotic signalling. Reduction in TBI-induced DNA damage accumulation coupled with inhibition of cell cycle arrest through stimulation of anti-p53- and anti-p21-dependent synergistic signalling protected ICPS cells from radiation injury. Enhanced proliferation of crypt stem cells, induction of anti-oxidant defence, subjugation of TBI-induced lipid peroxidation and phenotypic polarization of intestinal macrophages to anti-inflammatory M2 class underlie amelioration of RIII. Stimulation of multiple mitigative signalling processes by DAMTC appeared to be associated with enhanced protein acetylation, an important regulator of cellular responses to radiation damage. Our findings establish the mitigative potential of DAMTC against RIII by hyper-acetylation-mediated epigenetic regulation, which triggers axes of anti-apoptotic and pro-survival pathways, enabling proliferation and maintenance of ICPS cells leading to epithelial regeneration.

## Introduction

Total body-irradiation (TBI) leads to a pattern of events that result in the onset of acute radiation syndrome (ARS). Recent evidences from animal studies indicate that the gastro-intestinal damage is a major factor responsible for radiation-induced lethality in humans and other mammalian systems^1^. The classical gastro-intestinal (GI) injury occurs due to death of clonogenic crypt epithelial stem cells, which leads to enterocyte depletion, mucositis, secretory diarrhoea, mucosal barrier breakdown and system dysfunction involving intestinal elements viz. immune system, enteric muscularis, microvasculature, resident bacteria etc.^1,2^. Thus, the gastro-intestinal sub-syndrome of ARS (GI-ARS) is mainly characterised by apoptosis in the intestinal epithelium, intestinal bleeding, sepsis, electrolyte and fluid imbalance causing death^1^. Therefore, reconstitution of the intestinal epithelium is a pre-requisite for recovery and survival post GI-ARS.

In mammals, the GI syndrome arises from a TBI exposure of >6 Gy of low LET ionizing radiation like γ-rays and its pathophysiology includes protein modifications, alterations in redox status, inflammation-related secondary effects, cytokine release and impaired functionality due to cell loss^3^. Extensive vomiting and diarrhoea if not controlled exacerbate the fluid and electrolyte loss, culminating in death between 3–10 days^4^. Therefore, development of preventive and mitigative strategies to reduce radiation-induced GI-injury is a major constituent of medical countermeasures (MCMs) against ionizing radiation (IR)-induced ARS^1^.

The complex mechanisms of radiation-induced intestinal injury (RIII) constitute loss of clonogenic crypt cells, defective regeneration of intestinal stem cells, subsequent villus depopulation as well as a systemic inflammatory response syndrome post radiation exposure^1^. Survival following GI-ARS is primarily dependent on two factors viz. rate of depopulation of the crypts and efficiency of residual clonogens to regenerate crypt-villus units^5–7^. Consequently, management of RIII include administration of growth factors like interleukin-11 and keratinocyte growth factor, capable of promoting intestinal crypt cell proliferation and pre-treatment with transforming growth factor-β3 (TGF-β3) and β1 (TGF-β1), which help the regeneration of crypt cells^5–12^. Intestinal macrophages identify bacteria entering through the damaged intestinal mucosa and generate regenerative and repair signals that are transmitted to the epithelial progenitors in the ISC niche^8–12^. Stimulation of TLR (Toll-like Receptor) using various TLR ligands and agonists have been found to dampen intestinal sepsis, enhance intestinal crypt cell survival and regeneration thereby, improving animal survival following RIII^13–15^. It is now well established that initiation and development of IR-induced GI syndrome is caused by the death of intestinal crypt progenitor/ stem (ICPS) cell as well as vascular endothelial cells^16^. Therefore, approaches that reduce intestinal stem cell (Lgr5^+^) and ICPS cell apoptosis coupled with enhanced crypt proliferation and survival via prolonged anti-apoptotic NF-κB activation attenuate IR-induced intestinal injury and protect against RIII^16,17^.

Many strategies have been developed to combat GI-ARS, albeit none have gained approval by the FDA for human application. Thus, there is a compelling need for designing novel countermeasure agents to ameliorate IR-induced GI injury. Acetylation is one of the pivotal post-translational modifications (PTMs), which controls plethora of functions in cells including gene expression and chromatin remodelling by virtue of lysine acetyl transferases (KATs)^18^. The diverse biological and pharmacological benefits of many naturally occurring heterocyclic polyphenols are well established^19,20^. In addition to the widespread therapeutic benefits of the parent moiety, semi-synthetic acetyl derivatives of polyphenols viz. 7, 8-diacetoxy-4-methylcoumarin (DAMC) and 7-acetoxy-4-methylcoumarin (7-AMC) have been shown to participate in the acetylation of target proteins linked to a novel acetylation system referred to as acetoxy drug-calreticulin transacetylase (CRTase) system^21–26^. Some of the target proteins (enzymes) observed to be acetylated by the polyphenolic acetate (PA) DAMC, in a calreticulin-dependent manner viz. nitric oxide synthase (NOS), glutathione S transferase, thioredoxin, and NADPH-cyto-c reductase^27,28^ are well known modifiers of oxidative stress involved in the radiation response of cells^29,30^. More recently, DAMC has been shown to augment vascular endothelial growth factor (VEGF), thus, stimulating angiogenesis^29^ that could facilitate recovery from radiation damage. Thus, PAs appear to be potential candidates as radiation countermeasure agents. The sulphydryl containing PA, 7, 8- diacetoxy-4-methylthiocoumarin (DAMTC), is anticipated to reduce the oxidative stress more efficiently that the other acetylated polyphenols (like DAMC). By its acetyl group donating ability, DAMTC can regulate protein acetylation dynamics, thereby, impacting epigenetics and other damage response signalling events in the cell, thereby influencing intestinal repair and regeneration in response to radiation injury. Furthermore, epigenetic events such as chromatin modification by acetylation of both histone and critical non-histone proteins in the intestinal jejunum has important implications in triggering events favouring proliferation and replenishment of ICPS cells as well as in initiating repair and regeneration of the intestinal jejunum and can influence the radiation-induced GI-ARS. Our earlier studies have indeed established the mitigation potential of DAMTC against radiation-induced hematopoietic injury (H-ARS) in C57BL/6 mice that correlated well with the reduction in mortality^31^. Alleviation of TBI-induced myelosuppression, reversal of bone marrow suppression, restoration of blood cell indices, stimulation of stem cell repopulation from multiple lineages and immune modulation coupled with pro-inflammatory macrophage induction contributed to the mitigation of H-ARS^31^. Since, GI damage also contributes to the TBI-induced lethality, particularly at higher doses, we investigated the potential of DAMTC to mitigate TBI-induced GI injury in C57BL/6 mice when administered 24 hours following irradiation. Results show amelioration of GI-ARS by DAMTC and facilitation of recovery. Attenuation of IR-induced DNA damage and apoptotic cell death in the intestinal epithelium coupled with enhanced proliferation, stimulation of anti-oxidant defence reducing radiation-induced oxidative damage, and induction of anti-inflammatory (M2) macrophages by DAMTC appear to contribute to the mitigation of TBI-induced GI injury and associated mortality. Notably, DAMTC-mediated hyper-acetylation via novel acetoxy drug: CRTase appears to be a plausible mechanism underlying the observed effects of DAMTC in the intestinal epithelium following TBI, promoting ICPS replenishment and reconstitution of the intestinal jejunum leading to mitigation of GI-ARS.

## Methods

### Mice

Inbred C57BL/6 mice were obtained from the central experimental animal facility of the Institute. Female mice used in the study were aged 8–10 weeks, weighing 20–25 grams and were fed standard rodent feed (from Golden Feeds, Delhi, India) and water *ad libitum*. The facility housing mice cages was maintained at 23–25 °C, relative humidity of 55 ± 5% with a 12-hour light/12-hour dark cycle.

### Ethics statement

Animal studies were conducted with the approval from and in accordance with the guidelines and policies of the Committee on the Ethics of Animal Experiments, Institute of Nuclear Medicine and Allied Sciences (INMAS), Defence Research and Development Organization (DRDO) (Institutional Ethical Committee number under which this study has been approved is INM/IEAC/2011/08/001). Animals were euthanized and experiments were carried out using protocols approved by the Committee on the Ethics of Animal Experiments of INMAS, DRDO.

### Irradiation

Mice were subjected to total body irradiation (TBI) in ^60^Co Gamma Teletherapy unit (Bhabatron II, Panacea Medical Technologies, Bangalore, India) at a dose rate of 1 Gy/min (dose rate of the ^60^Co Gamma irradiation source was calibrated using physical dosimetry). All irradiations were carried out at room temperature. Evaluation of effects of DAMTC on TBI-induced GI-ARS was performed by exposing mice to sub-lethal dose of 7.6 Gy.

### Preparation and administration of 7, 8-diacetoxy-4-methylthiocoumarin (DAMTC)

Preparation and administration of DAMTC has been reported earlier^31^. Briefly, it was prepared fresh by dissolving in dimethyl sulfoxide (DMSO) and diluted in water (DMSO concentration <0.1%). Mice were intra-peritoneally administered 5 µg/kg of DAMTC at 24 hours post TBI. Total volume of drug administered per mice was 100 μl.

### Histological examination of the intestinal jejunum

Mice were euthanized and small intestine tissues (jejunum) were isolated on days 3, 7, 14 and 21 post treatment. Jejunal tissues were then fixed in 10% neutral-buffered formalin (SRL) overnight, dehydrated and embedded in paraffin. 5 µm-thick micro-sections were placed on slides and stained with hematoxylin and eosin (H&E). The slides were examined by light microscopy to capture bright-field images using Olympus (IX51) microscope (Japan). The intestinal sections were used for measurement of villus height, crypt depth and enumeration of crypts per field of view. For determination of crypt numbers ~5–7 field of view (FOV) per mouse/section were assessed. For villus height and crypt depth measurements numbers of villi and crypts assessed per animal were a minimum of 30–35 villi and at-least 40–45 crypts respectively (per mouse).

### Assessment of intestinal functionality by xylose absorption assay

Xylose uptake assay was performed to determine functional regeneration of intestinal absorption as a physiological indicator of mucosal barrier integrity as described earlier^32^ at days 3, 7 and 10 following treatment. Briefly, mice were administered with 5% w/v solution of D-xylose (100 μl) orally. Minimal amount of blood (~150–200 μl) was withdrawn 2 hours post D-xylose administration from the retro-orbital plexus of mice. The blood containing vials were placed undisturbed in a slanting position for about 1 hour at room temperature and centrifuged at 6000 rpm for 20 minutes at 4° C to isolate serum. 50 μl of serum sample was mixed with 5 ml of phloroglucinol reagent (0.5 g phloroglucinol, 100 ml glacial acetic acid and 10 ml concentrated HCl) and placed in a water bath at 100° C for 5 minutes. The absorbance of samples at a wavelength of 554 nm was measured on a spectrophotometer.

### Terminal deoxynucleotidyl transferase dUTP nick end labeling (TUNEL) assay to label DNA breaks for apoptosis determination by immunohistochemistry

Apoptosis was assessed in the jejunal sections from mice at days 3, 7, 14 and 21 post treatment, using ApoBrdU-IHC DNA fragmentation assay kit (RayBiotech, Inc.) according to the manufacturer’s instructions. Briefly, paraffin-embedded sections were deparaffinised, rehydrated and incubated with the labelling reaction mixture. After completion of the assay, the slides were examined by light microscopy to capture bright-field images using Olympus (IX51) microscope (Japan).

### Western immunoblotting analysis in the intestinal jejunum

Mice jejuna were dissected out post euthanasia on days 3, 7 and 21 following treatment. The jejunal tissues were incubated in ice-cold lysis buffer (RIPA) with 1 mM phenyl-methylsulfonyl fluoride (PMSF) and complete protease inhibitor cocktail (Sigma, USA) on ice and homogenized to extract protein. The protein concentrations were quantified using BCA protein assay kit (Thermo Scientific, USA). Samples containing equal amounts of protein (50 μg) were resolved using 8–15% SDS-PAGE gel and transferred onto polyvinylidene difluoride (PVDF) membranes (Amersham) as described earlier^33^. The membranes were subjected to overnight incubation at 4° C with primary antibody against PCNA, Caspase-3, PARP, NF-κB, p53, p21, Cyclin D1, Cyclin B1, Catalase, SOD-2, acetylated-histone H3 (K9/14), β-actin (Santa Cruz Biotechnology, Inc., USA) and Bcl-2, Bax, phospho-CHK2 and Arginase-1 (Cell Signaling Technologies, USA). The membranes were then incubated with appropriate horseradish peroxidase-conjugated secondary antibody at room temperature. The proteins were detected with a chemiluminescent substrate and signal captured using Michrochemi (DNR Bioimaging Systems, Israel).

### Lipid peroxidation assessment in the mouse jejunum by thio-barbituric acid reactive substances (TBARS) assay

Mice were euthanized and jejuna isolated on days 3, 7, 14 and 21 post treatment. The tissue was homogenized in ice-cold lysis buffer on ice and TBARS assay was performed in the tissue homogenate essentially as described earlier^34^. Briefly, samples were mixed with reaction buffer, tri-chloroacetic acid (TCA) and TBA, followed by incubation at 80° C for 45–50 minutes. The samples were immediately placed on ice after the incubation for 10 minutes and sample absorbance was recorded at 532 nm using a spectrophotometer. MDA, the end-product of lipid peroxidation in the jejunal tissue is expressed in nmoles/mg tissue.

### Statistical analysis

Data analysis was done using Graph Pad Prism (version 5.01) and is represented as mean ± standard error of mean (SEM). Significance of difference between groups was calculated by Student’s t test and One-way or Two-way ANOVA with Tukey’s, Dunnett’s, Newman-Keuls’ or Bonferroni’s multiple comparison post-tests. Results were considered to be significant at P < 0.05.

## Results

### DAMTC mitigates TBI-induced structural injury in intestine

**Figure 1:**
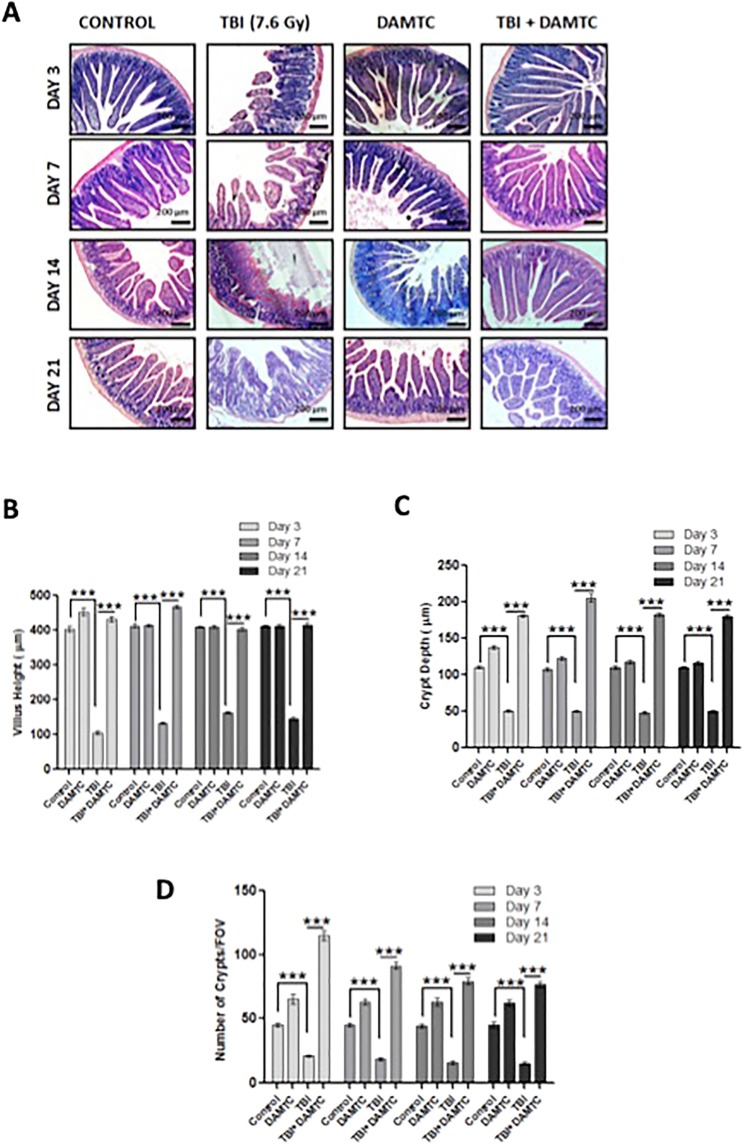
DAMTC mitigates TBI-induced intestinal structural injury in mice. Effects of DAMTC on intestinal structural integrity in TBI mice by histological examination. (**A**) Micrographs show H&E staining of mouse small intestinal sections. Representative images are shown for naïve, DAMTC, TBI (7.6 Gy), and TBI + DAMTC groups at days 3, 7, 14 and 21 following TBI and treatment. Scale bar = 200 μm with an original magnification of x100. (**B**) Villus height. (**C**) Crypt depth and (**D**) crypt numbers per field of view (FOV) seen in naïve, DAMTC, TBI (7.6 Gy), TBI + DAMTC mice (intestinal sections from ten animals were examined in each group; n = 10). All error bars indicate SEM. *P < 0.05; **P < 0.01; ***P < 0.001.

The putative role of DAMTC in combating RIII was assessed by studying histological changes (gross morphology) within the small intestine (jejunum). The end points for assessing radiation-induced intestinal structural injury include measurement of crypt depth, villus height and scoring number of crypts per field^17^. Destruction of Intestinal jejunum architecture and epithelial structural disorganization were visualized as crypt atrophy and villus sloughing in the TBI cohort as early as day 3 post TBI (Fig. 1A). The crypt-villi structures of small intestine were preserved in the DAMTC-TBI mice at days 3, 7, 14 and 21 post TBI (Fig. 1A). On the contrary, structural architecture of the damaged intestinal tissue was not restored to normalcy even at day 21 in the TBI mice (Fig. 1A). Additionally, we measured the crypt depth, villus height and enumerated crypts per field in the intestinal tissues from different treatment cohorts as indices of structural integrity. A significant reduction in villus height was evident following TBI at days 3 (105.5 ± 8.65 μm; p < 0.0001), 7 (133.7 ± 2.6 μm; p < 0.0001), 14 (162.7 ± 3.18 μm; p < 0.0001) and 21 (144.3 ± 4.842 μm; p < 0.0001) compared to the sham-irradiated (naive/control) mice (day3, 403.3 ± 8.65 μm; day 7, 413.7 ± 6.57 μm; day 14, 411.0 ± 3.06 μm; day 21, 413.0 ± 2.74 μm) (Fig. 1B). DAMTC treatment alleviated the radiation-induced villus denudation, evident from the increment in the villus height at days 3, (433 ± 7.54 μm; p < 0.0001), 7 (468.3 ± 4.33 μm; p < 0.0001), 14 (404 ± 5.18 μm; p < 0.0001) and 21 (416.3 ± 7.33 μm; p < 0.0001) (Fig. 1B). Altered crypt morphology was also noted as a decline in the crypt size/depth following RIII. Crypt depth was drastically (>2 fold) reduced as early as day 3 following TBI and remained significantly lower compared to the un-irradiated mice (p < 0.0001), which was reversed by DAMTC (p < 0.0001) at all observation times (Fig. 1C). Concomitant to the destruction of villus and shortening of crypts, enormous decrease in numbers of crypts per field was visualized in the TBI cohort at all the evaluated post-irradiation time points when compared with the naive (p < 0.0001) and DAMTC-TBI mice (p < 0.0001) (Fig. 1D). Taken together, these findings indicate that DAMTC ameliorates radiation injury in the intestine (GI-ARS) of mice by enabling structural reconstitution.

### Recovery of intestinal absorption and restoration of functionality

**Figure 2:**
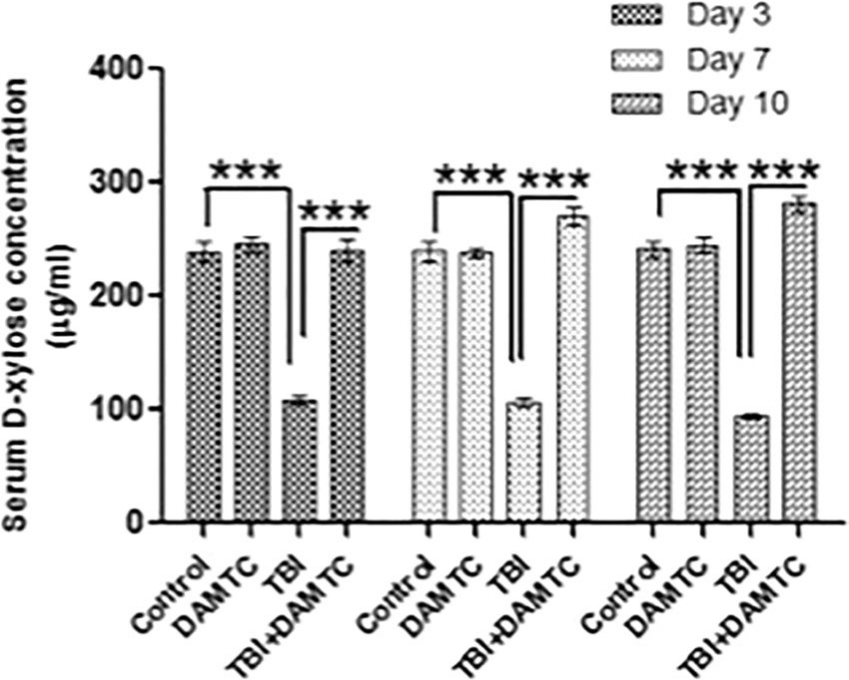
DAMTC restores intestinal functionality in TBI mice. Effects of DAMTC on intestinal functional regeneration in TBI mice by Xylose absorption assay. D-Xylose absorption for naïve, DAMTC, TBI, and TBI + DAMTC groups at days 3, 7 and 10 following TBI (ten animals were examined in each group; n = 10). All error bars indicate SEM. *P < 0.05; **P < 0.01; ***P < 0.001.

To determine the functional regeneration and absorptive capacity of the intestine following TBI and DAMTC treatment, we performed the xylose absorption assay. Since, D-Xylose is a non-metabolizable sugar in the body, serum xylose levels provide a reliable insight into the functional absorptive efficacy of the intestine^35^. Intestinal absorption of D-Xylose was significantly impaired by irradiation at day 3 (108.8 ± 3.88 μg/ml), when compared with sham-irradiated mice (239.3 ± 9.72 μg/ml; p < 0.0001) (Fig. 2). Improvement in the intestinal absorptive capacity of TBI mice was absent at days 7 (106.0 ± 4.57 μg/ml; p < 0.0001) and 10 (94.4 ± 2.71 μg/ml; p < 0.0001). In contrast, a significant recovery in the xylose absorption was evident in DAMTC-TBI mice from day 3 (240.3 ± 9.14 μg/ml; p < 0.0001), reaching baseline control levels (Fig. 2). Furthermore, progressive improvement in the serum xylose levels was exhibited by the DAMTC-TBI cohort at day 7 (270.4 ± 8.28 μg/ml; p < 0.0001) and sustained levels of serum xylose were noted at day 10 (281.4 ± 7.42 μg/ml; p < 0.0001) (Fig. 2). These findings suggest that DAMTC facilitates rapid restitution of the intestinal villi, leading to recovery in the absorptive capacity and restoration of the intestinal functional potential following TBI.

### Inhibition of radiation-induced apoptosis in the intestinal jejunum

**Figure 3:**
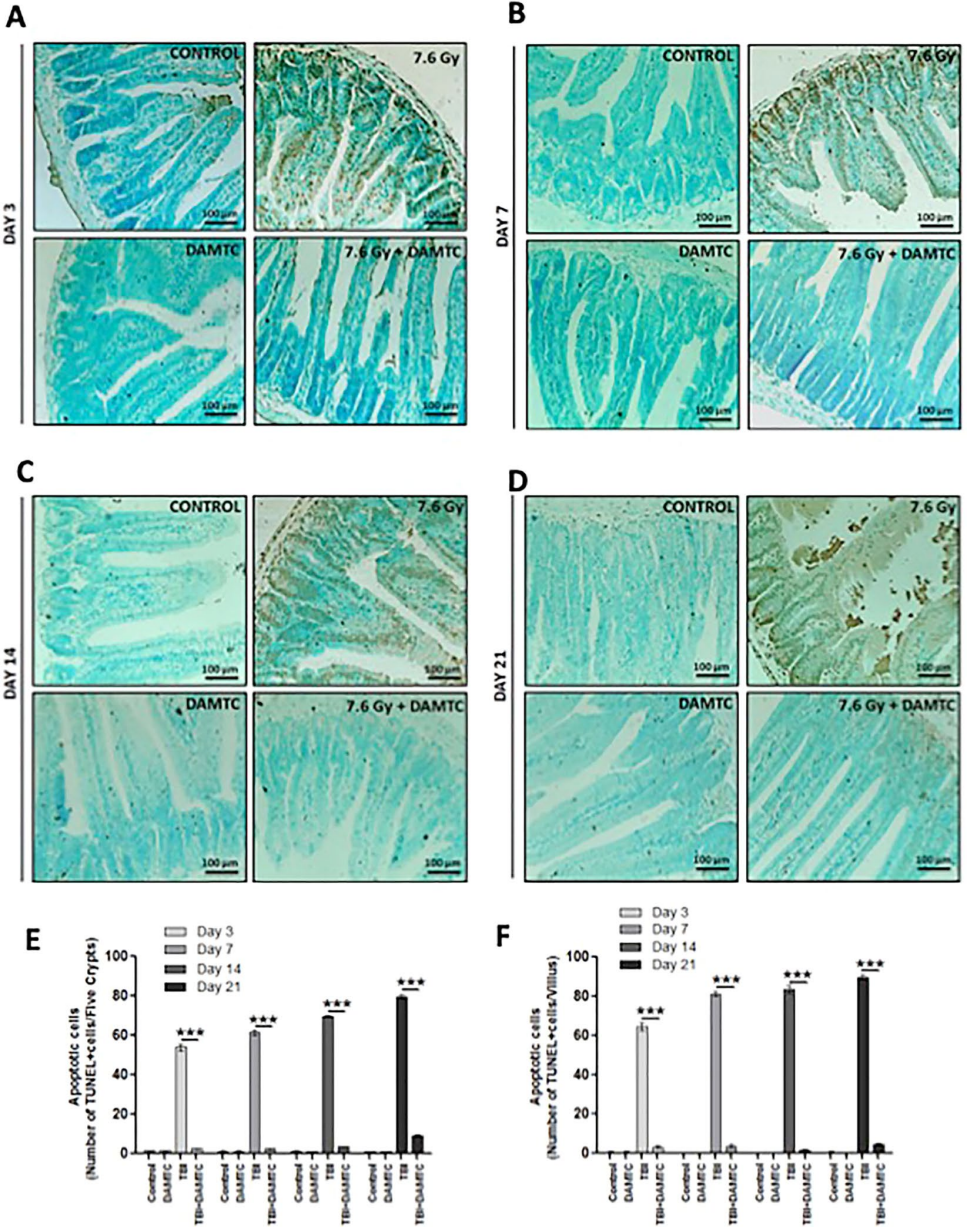
DAMTC mitigates TBI-induced apoptosis in the intestinal crypt epithelium and villus of mice. Effects of DAMTC on apoptosis in TBI mice was evaluated by TUNEL staining (brown) (counter-stain: methyl green). Micrographs show TUNEL staining of mouse small intestinal sections. Panels show representative images for naïve, DAMTC, TBI (7.6 Gy), and TBI + DAMTC groups at days (**A**) 3, (**B**) 7, (**C**) 14 and (**D**) 21 following TBI. Scale bar = 100 μm with an original magnification of x200. Enumeration of (**E**) apoptotic cells per five crypts and (**F**) apoptotic cells per villus (intestinal sections from ten animals were examined in each group; n = 10). All error bars indicate SEM. *P < 0.05; **P < 0.01; ***P < 0.001.

Since ionizing radiation (IR) induces apoptosis of intestinal crypt epithelial cells and stromal cells in the villus^35–37^, biochemical changes related to cell death were investigated. Apoptotic cell death was assessed by performing Terminal deoxynucleotidyl transferase dUTP nick end labelling (TUNEL) staining of intestinal tissue sections at days 3, 7, 14 and 21 following TBI in mice (Fig. 3A–D). Intestinal epithelium is known to be replenished by the stem and progenitor cells that cohabitate in the crypts^38–40^. TUNEL-reactive enterocyte nuclei enumeration as a measure of apoptotic cell death, revealed abundance in apoptotic cells in crypt and villus of TBI mice (Fig. 3E,F). DAMTC decreased the radiation-induced TUNEL-positive cells, corresponding to the intestinal crypt progenitor/stem cells (ICPS) by nearly 22 fold (2.52.5 ± 0.29 vs 54.0 ± 1.83; p < 0.0001) in the jejunal crypts, at day 3 post TBI (Fig. 3E). A similar pattern in fold difference of number of apoptotic nuclei in crypt epithelium was observed at days 7 and 14 post TBI. DAMTC treatment caused a robust decrease in apoptotic ICPS cells by nearly 25 fold (2.5 ± 0.29; p < 0.0001) and 20 fold (3.4 ± 0.4; p < 0.0001) at days 7 and 14 respectively over TBI mice (day7, 61.5 ± 1.26; day 14, 69.5 ± 0.65). At day 21, TBI cohort exhibited about 9 fold greater TUNEL-positive cells in the intestinal crypts (79.6 ± 0.81; p < 0.0001), which was drastically reduced by DAMTC (9.0 ± 0.45) (Fig. 3E). A concomitant and significant accretion in apoptotic stromal cells in the villus was apparent in TBI mice at all the aforementioned times. At days 3, 7 and 21, an enormous (over 20 fold) increase in the number of apoptotic cells in the intestinal villus was evident in TBI mice (64.5 ± 2.06 at day 3, p < 0.0001; 81.25 ± 1.25 at day 7, p < 0.0001; 89.75 ± 1.18 at day 21, p < 0.0001) as compared with DAMTC-TBI mice (3.25 ± 0.63 at day 3; 3.75 ± 0.63 at day 7; 4.6 ± 0.51 at day 21) (Fig. 3F). DAMTC treatment profoundly decreased (~48 fold) the TUNEL-positive cells in the villus stroma of TBI mice (83.5 ± 1.94 vs 1.75 ± 0.48; p < 0.0001) at day 14 (Fig. 3F). These observations are consistent with earlier findings^37–39^, showing that apoptosis of ICPS as well as villus stromal cells contribute towards RIII following TBI. Thus, DAMTC alleviates TBI-induced apoptosis in the intestinal jejunum, which may contribute towards the mitigation of GI injury in mice.

### Reduction in TBI-induced intestinal apoptosis by abrogation of apoptotic pathway

**Figure 4:**
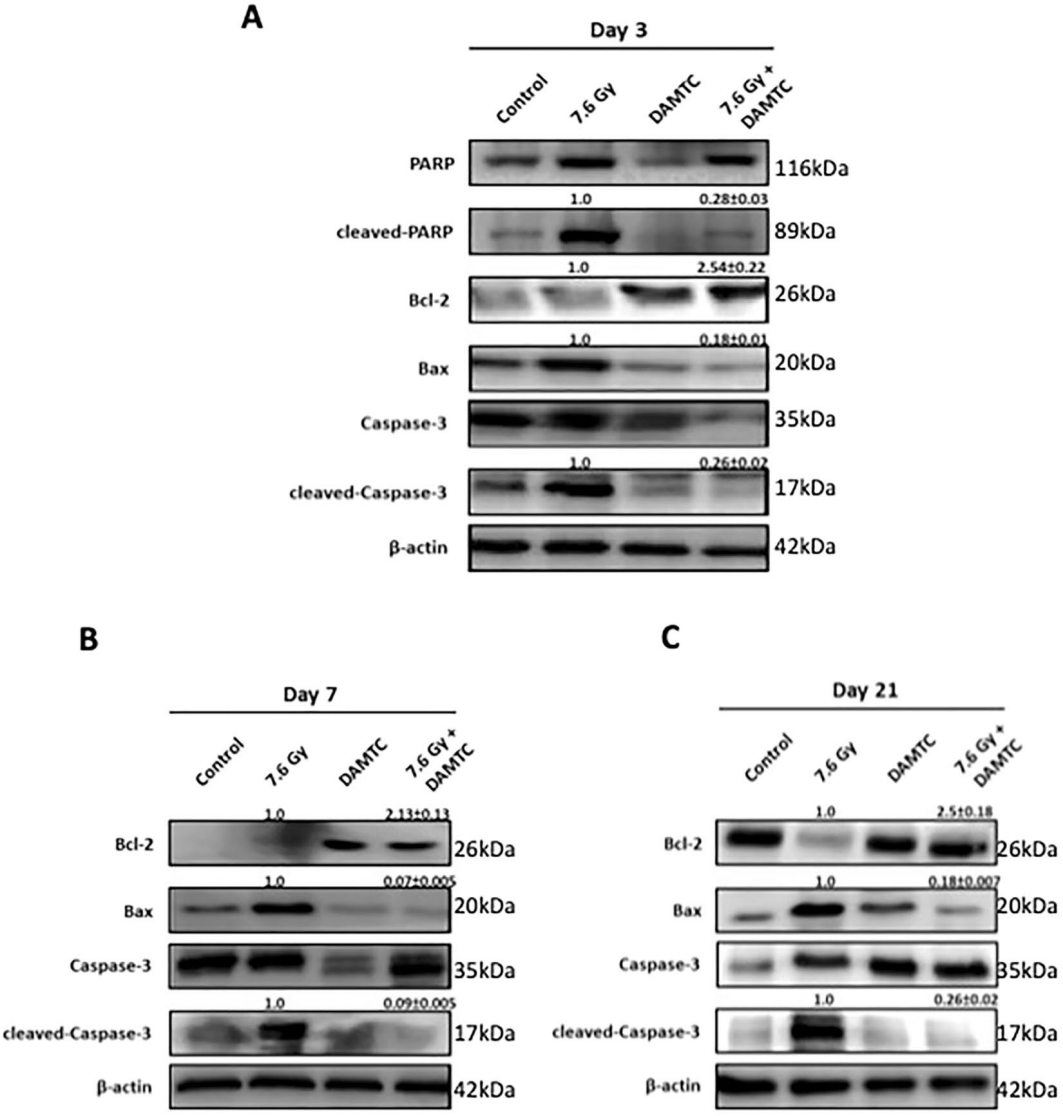
DAMTC mediates abrogation of apoptotic pathway in the intestine of TBI mice. Effects of DAMTC on radiation-induced apoptosis in TBI mice by immunoblotting analysis of pro-apoptotic (Bax, cleaved-caspase3, cleaved-PARP) and anti-apoptotic (Bcl-2) proteins in the intestinal jejunum. Changes in levels of the aforementioned proteins for naïve, DAMTC, TBI (7.6 Gy), and TBI + DAMTC mice at days (**A**) 3, (**B**) 7 and (**C**) 21 following TBI. Blots represented are from the same gel and β-actin served as the loading control. Membrane stripping to re-probe for a different protein was done wherever required. Quantifical analysis of immunoblots represent average relative fold change in protein levels (normalized to β-actin) with SEM between TBI (7.6 Gy) and TBI + DAMTC groups (from five independent observations).

Caspase-3 plays a central role in the execution of radiation-induced apoptosis, causing degradation of chromosomal DNA within the nucleus coupled with chromatin condensation^41,42^. Therefore, we investigated the expression of caspase-3 to examine the effect of DAMTC on TBI-induced apoptosis pathway in the mouse intestine (jejunum). A notable decrease in the levels of active caspase-3 was evident in mice that received DAMTC following irradiation as compared with their TBI counterparts (p < 0.0001) (Fig. 4A,B,C). Active caspase-3 is known to cause proteolytic cleavage and activation of several key proteins such as poly (ADP-ribose) polymerase (PARP), that eventually mediate the biochemical events and morphological changes seen in the apoptotic cells^43^. In accordance with increased expression of active caspase-3, a concomitant augmentation in the expression of active PARP/cleaved-PARP (cPARP) was observed at day 3 in the TBI mice (Fig. 4A). However, cPARP level was negligible in DAMTC-TBI mice at day 3 (p < 0.0001), suggesting the inhibition of apoptosis and DNA fragmentation and alleviation of radiation injury in the jejunum as early as day 3 post TBI (Fig. 4A). We then examined the levels of Bax and Bcl-2, the pro-apoptotic and anti-apoptotic proteins respectively, that serve as upstream regulators of caspase activity and apoptotic process^44^. Enhanced apoptosis was confirmed by increased Bax expression on days 3, 7 and 21, that correlated with increased active caspase-3 levels in the TBI mice (Fig. 4A–C). In contrast, apoptotic suppression in DAMTC-TBI mice was further corroborated by decreased Bax expression at the aforementioned time points (p < 0.0001). An elevation in Bcl-2 level visualized under these conditions was in conformity with the down-regulation of apoptotic process in DAMTC-treated mice (Fig. 4A–C), as compared to a decrease in Bcl-2 level in the TBI mice indicative of apoptotic stimulation (Fig. 4A–C) (p < 0.001). These findings suggest that DAMTC induces Bcl-2 family-mediated anti-apoptotic signalling in the intestinal jejunum, which appears to facilitate mitigation of GI-ARS by subjugation of TBI-induced programmed cell death in C57BL/6 mice.

### Augmentation of cell proliferation and activation of pro-survival signalling

**Figure 5:**
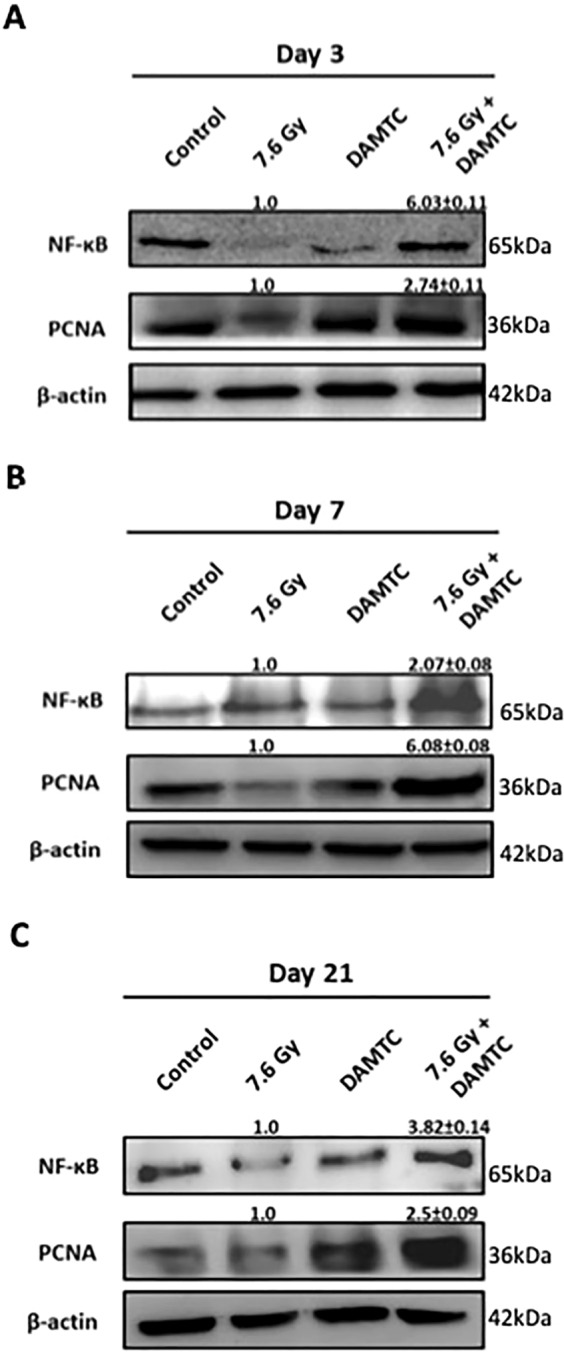
DAMTC facilitates activation of pro-survival signalling and augmentation of cell proliferation in the intestine of TBI mice. Effects of DAMTC on cell proliferation in TBI mice by immunoblotting analysis of NF-κB and PCNA in the intestinal jejunum. Changes in the protein levels of naïve, DAMTC, TBI (7.6 Gy), and TBI + DAMTC mice at days (**A**) 3, (**B**) 7 and (**C**) 21 following TBI. Blots represented are from the same gel and β-actin served as the loading control. Membrane stripping to re-probe for a different protein was done wherever required. Quantifical analysis of immunoblots represent average relative fold change in protein levels (normalized to β-actin) with SEM between TBI (7.6 Gy) and TBI + DAMTC groups (from five independent observations).

Sustenance and maintenance of the intestinal jejunum is attributed to the resident intestinal crypt stem (ICS) cells, which serve to replenish constantly the epithelial cells that are annihilated and lost from the villus^5,40^. Epithelial cell renewal is crucial for re-establishment of the jejunum architecture post structural disorganisation caused by RIII^38,40^, underscoring the importance of ICS cells to ensure sustained recovery from TBI-mediated GI-ARS^45,46^. Severity of RIII is exacerbated by sustained apoptosis of ICS and their impaired regeneration^45,46^. In the present study, we noted a profound increase in the apoptotic death of the ICPS cells following TBI in mice, which was reversed by DAMTC administration (Fig. 3). Additionally, we determined the effect of DAMTC on the regeneration of the ICS cells by studying expression of proliferating cell nuclear antigen (PCNA) in the jejunum. PCNA, identified originally as a co-factor of DNA polymerase δ, plays an essential role in DNA synthesis/replication and is regarded as a critical marker for cell proliferation^42^. Under these conditions, proliferative capacity of ICS cells was enhanced by DAMTC in TBI mice, as evident from the enrichment in PCNA levels (Fig. 5A–C) (p < 0.0001). Since, PCNA is a proliferation marker and the ICS cells majorly constitute the proliferating component of the intestinal jejuna, the levels of PCNA are expected to be proportional to the proliferating ability/status of the ICS cells. Further, we found a marked escalation in NF-κB levels in the mouse jejunum on DAMTC administration in TBI mice at days 3, 7 and 21 (p < 0.0001) (Fig. 5A–C). Taken together, these results demonstrate the efficacy of DAMTC in stimulating proliferation of cycling (mostly ICS) cells in the intestinal jejunum following TBI, in addition to the prolonged survival of these cells to combat radiation injury in mice.

### Attenuation of radiation-induced DNA damage and facilitation of cell cycle progression

**Figure 6:**
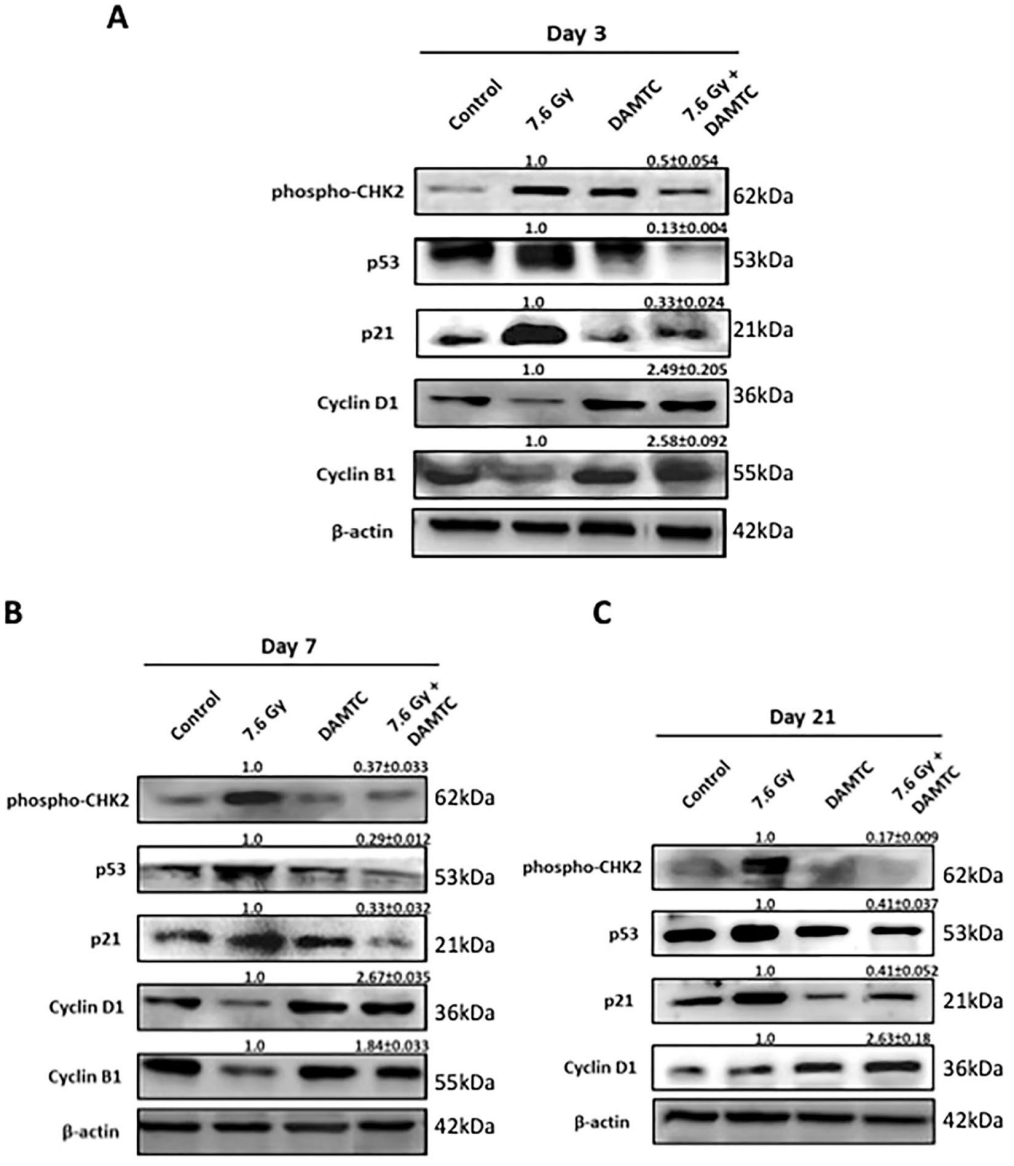
DAMTC mitigates radiation-induced DNA damage accumulation and facilitates cell cycle progression in the intestine of TBI mice. Effects of DAMTC on DNA damage in TBI mice studied by immunoblotting analysis of DNA damage pathway proteins (phospho-CHK2, p53, p21) and cell-cycle checkpoint proteins (cyclins) in the intestinal jejunum. Changes in the protein levels in naïve, DAMTC, TBI (7.6 Gy), and TBI + DAMTC mice at days (**A**) 3, (**B**) 7 and (**C**) 21 following TBI. Blots represented are from the same gel and β-actin served as the loading control. Membrane stripping to re-probe for a different protein was done wherever required. Quantifical analysis of immunoblots represent average relative fold change in protein levels (normalized to β-actin) with SEM between TBI (7.6 Gy) and TBI + DAMTC groups (from five independent observations).

IR-induced DNA damage causes delayed progression of cells through the cell cycle and/or subsequent cell cycle arrest, prior to activation of programmed cell death^47–49^. CHK2 kinase is one of the DNA damage-induced check point effector molecules activated by ATM kinase^50,51^, implicating phosphorylated CHK2 (pCHK2) as a radiation-responsive DNA damage biomarker^51^. Analysis of the intestinal jejunum cells revealed higher levels of p53 (damage linked stabilization) (p < 0.0001) and pCHK2 at days 3, 7 and 21 post TBI (p < 0.0001) suggestive of pronounced DNA damage response (DDR), while their levels were consistently lower in DAMTC-TBI mice (Fig. 6A–C) indicating a lower level of DNA damage. These observations suggest that DAMTC profoundly suppresses radiation-induced p53 activation and apoptosis, thereby, influencing the survival of ICS cells by ameliorating TBI-induced DNA damage in the epithelial crypt stem cells. Furthermore, these findings are in line with, and corroborate our observations showing attrition of radiation-induced ICPS cell death by DAMTC (Fig. 3). Following IR exposure and induction of DNA damage, transcriptionally active p53 triggers p21 expression, an inhibitor of CDK2 and CDK4 activity, which eventually arrests the cell cycle at G1/S and functions in the maintenance of G2/M checkpoint in few cells^52–54^. Further, increased p21 expression in the intestinal crypts enhances the susceptibility of the ICPS cells to IR-induced cell death^54^. DAMTC down-regulated the IR-induced expression of p21 in the intestinal jejunum of TBI mice at all the observed time intervals (days 3, 7 and 21; Fig. 6A–C) (p < 0.0001). These results suggest that DAMTC elicits an anti-p53- and anti-p21-dependent synergistic signal to protect and replenish the proliferating (mostly the ICPS) cells, to offset GI-ARS by inhibition of TBI-mediated DNA damage accumulation, cell cycle arrest and apoptosis in the intestinal jejunum. Additionally, DAMTC augmented expression of cyclin D1 (p < 0.0001) and cyclin B1 (p < 0.0001) in TBI mice (Fig. 6A–C), lending support to the proposition that DAMTC prevents the causation of delayed DNA damage post TBI, facilitating the unperturbed progression of proliferating cells through the cell cycle to preserve and refurbish the intestinal jejunum. Cumulatively, these findings reveal the remarkable potency of DAMTC in markedly reducing the extent of TBI-mediated DNA damage accumulation in the intestinal jejunum to mitigate GI-ARS.

### Amelioration of radiation-induced oxidative stress and acceleration of antioxidant defence mechanism

**Figure 7:**
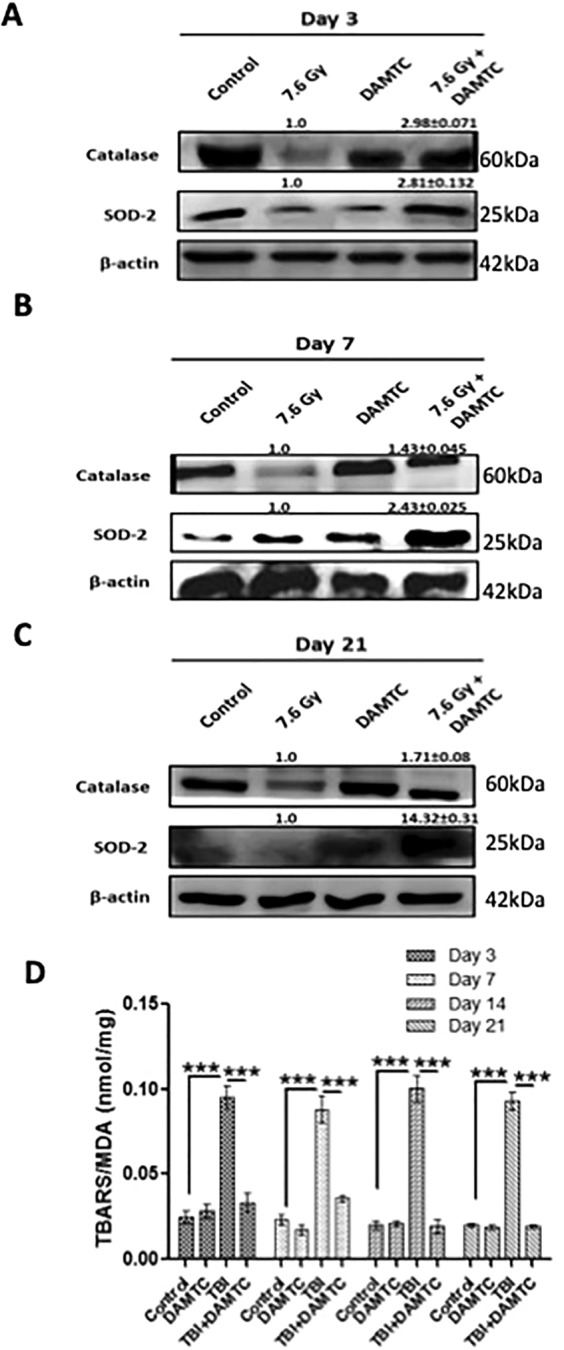
DAMTC induces anti-oxidant defence to reduce radiation-induced oxidative stress in the intestine of TBI mice. Effects of DAMTC on oxidative stress in TBI mice by immunoblotting analysis of anti-oxidant proteins (catalase and SOD-2) in the intestinal jejunum. Dynamics of molecular changes in naïve, DAMTC, TBI (7.6 Gy), and TBI + DAMTC mice at days (**A**) 3, (**B**) 7 and (**C**) 21 following TBI and treatment. Blots represented are from the same gel and β-actin served as the loading control. Membrane stripping to re-probe for a different protein was done wherever required. Quantifical analysis of immunoblots represent average relative fold change in protein levels (normalized to β-actin) with SEM between TBI (7.6 Gy) and TBI + DAMTC groups (from five independent observations). (**D**) Evaluation of lipid peroxidation by TBARS assay and MDA production in naïve, DAMTC, TBI (7.6 Gy), and TBI + DAMTC mice (n = 10 for each group). All error bars indicate SEM. *P < 0.05; **P < 0.01; ***P < 0.001.

IR-mediated oxidative tissue injury is initiated by radio-lytic hydrolysis of water and generation of reactive oxygen species (ROS)^55,56^. The highly reactive free radicals cause considerable damage to cellular macromolecules by resulting in carbonylation of proteins, lipid peroxidation within membranes and DNA damage^55,56^. DAMTC-dependent activation of anti-oxidant defence was evident from the elevated expression of anti-oxidant enzymes SOD-2 (p < 0.0001) and catalase (p < 0.001) in the intestinal tissue at all the post-irradiation times studied (Fig. 7A–C). Similarly, the TBI-induced higher levels of malondialdehyde (MDA) in the jejunal tissue was nearly abolished by DAMTC (p < 0.0001) at 3, 7, 14 and 21 days post irradiation (Fig. 7D). These observations indeed display DAMTC-mediated activation of anti-oxidant defence post TBI facilitating the reversal of TBI-induced heightened intrinsic oxidative injury in the intestinal jejunum to mitigate GI-ARS in mice.

### Epigenetic regulation by modulation of acetylation

**Figure 8:**
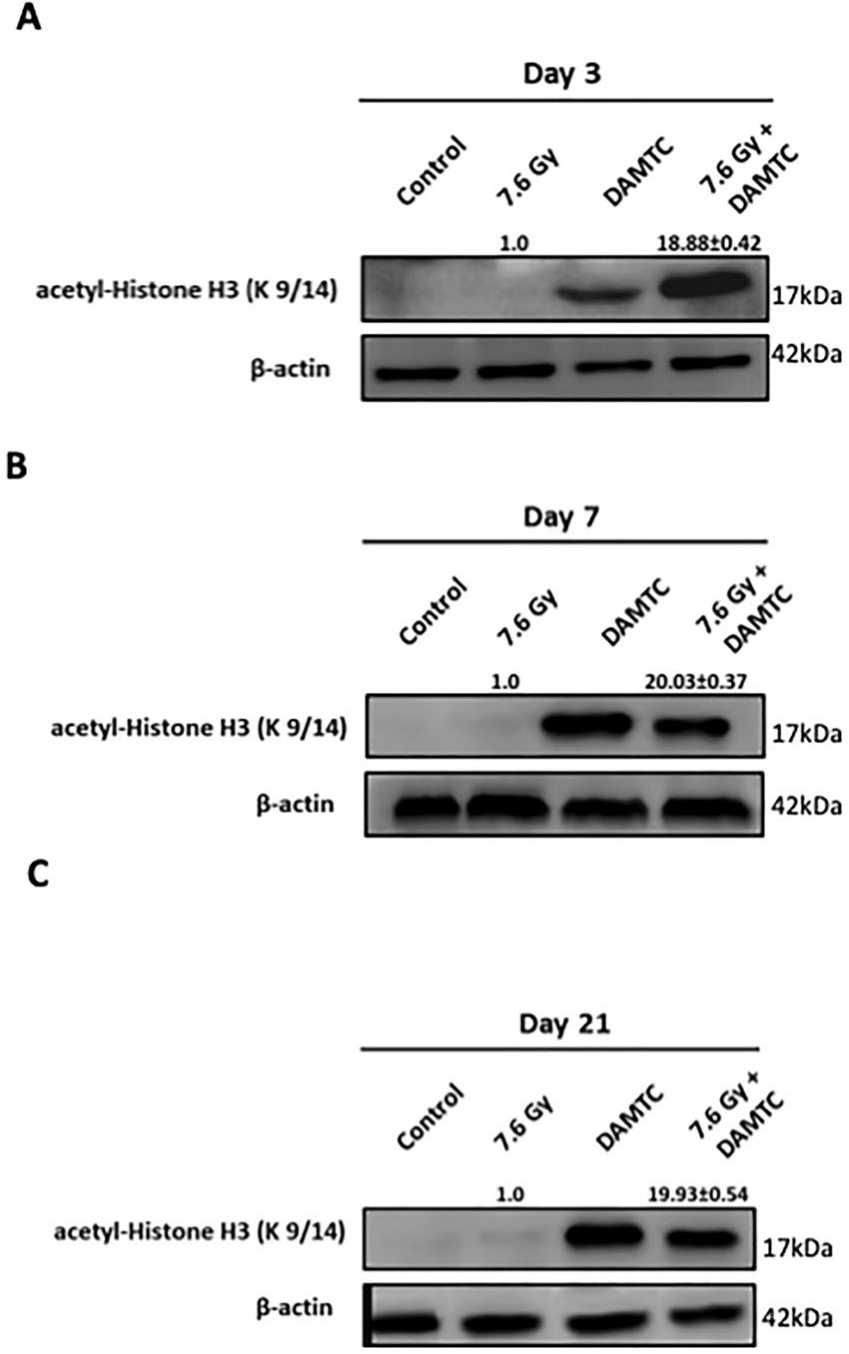
Epigenetic regulation via acetylation by DAMTC to mitigate GI-ARS in TBI mice. Effects of DAMTC on acetylation in TBI mice by immunoblotting analysis of acetyl-Histone H3 (at lysine 9/14) in the intestinal jejunum. Changes in the protein levels of naïve, DAMTC, TBI (7.6 Gy), and TBI + DAMTC mice at days (**A**) 3, (**B**) 7 and (**C**) 21 following TBI. Blots represented are from the same gel and β-actin served as the loading control. Membrane stripping to re-probe for a different protein was done wherever required. Quantifical analysis of immunoblots represent average relative fold change in protein levels (normalized to β-actin) with SEM between TBI (7.6 Gy) and TBI + DAMTC cohorts (from five independent observations).

Polyphenolic acetates (PAs) mediate protein acetylation by virtue of a novel acetoxy drug: calreticulin transacetylase system (CRTase)^21,23,24,27,28^ and have been shown to cause hyper-acetylation of low molecular weight proteins such as histones^57^. Histone acetylation has several implications including chromatin remodelling, altered gene expression, role in DNA replication and activation of key signal transduction cascades relevant in a cell-context specific manner^58^. We examined whether the mitigation of radiation damage by DAMTC is linked to the alterations in the acetylation status of histone H3 (H3 lysine 9/14) in TBI mice (Fig. 8). DAMTC profoundly increased the levels of acetylated-H3 in the intestinal jejunum following TBI (Fig. 8A–C) (p < 0.0001). These observations suggest that hyper-acetylation of H3 is mediated by DAMTC and appears to be an important contributing factor in altering the cellular responses to IR and mitigation of GI-ARS in mice.

### Induction of M2 macrophages and tissue repair

**Figure 9:**
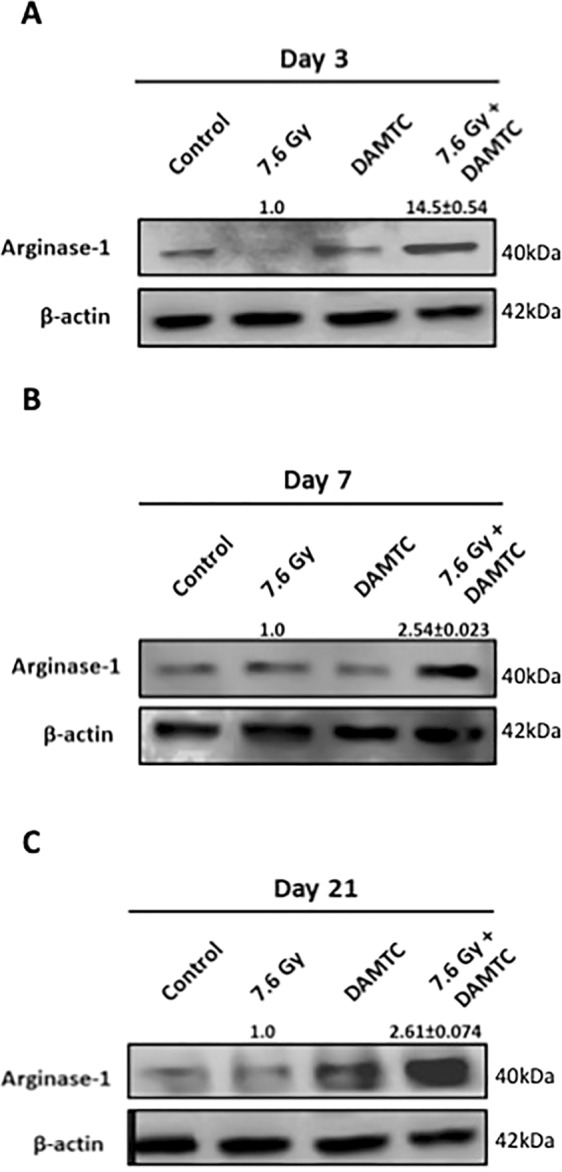
DAMTC facilitates induction of M2 macrophages in the intestinal jejunum of TBI mice. Effects of DAMTC on macrophage polarization in TBI mice by immunoblotting analysis of M2 macrophagic marker (Arginase-1) in the intestinal jejunum. Changes in the protein levels of naïve, DAMTC, TBI (7.6 Gy), and TBI + DAMTC mice at days (**A**) 3, (**B**) 7 and (**C**) 21 following TBI. Blots represented are from the same gel and β-actin served as the loading control. Membrane stripping to re-probe for a different protein was done wherever required. Quantifical analysis of immunoblots represent average relative fold change in protein levels (normalized to β-actin) with SEM between TBI (7.6 Gy) and TBI + DAMTC groups (from five independent observations).

The intestine possesses the largest compartment of the immune system owing to its greatest susceptibility to infections in the body^59^. Mononuclear phagocytes such as macrophages constitute one of the most abundant leucocytes in the intestinal jejunum and play a key role in combating infection and maintaining tissue homeostasis^59^. Furthermore, macrophages undergo phenotypic polarization in response to local tissue cues^59^, thereby exerting heterogeneous effects. Arginase-1 (Arg-1) is known to be predominantly expressed by tissue macrophages and these M2 macrophages exhibiting Arg-1 expression are known to create an anti-inflammatory environment that facilitate resolution of inflammation, cell proliferation and tissue repair, besides suppressing Th2-dependent inflammation^60^. DAMTC enhanced the Arg-1 expression in the intestinal jejunum of TBI mice at all evaluated time intervals (p < 0.0001) (Fig. 9A–C). Arginase-1 is expressed in different tissues and cell types like hepatocytes, intestinal cells, smooth muscle cells etc., wherein it is predominantly expressed, highly upregulated and induced in the macrophages^59^. Our observations therefore, provide a preliminary insight into the possible polarization of intestinal macrophages to M2 phenotype induced by DAMTC in TBI mice. Further studies on macrophage phenotyping in the intestinal jejunum of mice following TBI as well as key signalling events regulating immune-modulation patterns triggered by DAMTC post TBI are warranted.

## Discussion

Injuries to the gastro-intestinal and hematopoietic systems contribute to the lethality of TBI at low to moderate doses of radiation^1,4^. Radiation-induced GI injury mainly arises from massive apoptosis in intestinal epithelium as a consequence of mucosal barrier disintegration, electrolyte imbalance, sepsis and intestinal bleeding^1,2,4^. Enhanced survival of the progenitors coupled with mobilization and proliferation of surviving intestinal crypt stem cells following TBI to replenish the stem cell pool are the two chief factors contributing towards reconstitution of the GI system, promoting intestinal function and animal survival. Our results show that DAMTC minimises TBI-induced apoptosis in the jejunum (cycling ICPS and villus stromal cells) (Fig. 3), stimulates crypt stem cell proliferation (Fig. 5), enabling intestinal repopulation and regeneration, besides reducing the accumulation of DNA damage (Fig. 6) coupled with enhanced anti-oxidant defence (Fig. 7), which collectively contribute to the mitigation of radiation-induced GI injury. Mitigation of hematopoietic injury by DAMTC when administered 24 hours after exposure to lethal TBI as reported by us earlier^31^ and mitigation of GI-ARS demonstrated here make DAMTC a strong candidate as a practically useful radiation countermeasure agent for human application in instances of planned as well as accidental radiation exposure.

Disintegration of mucosal barrier, intestinal ablation and immune-suppression and enterocyte depletion are the manifestations of GI-ARS^1,2,4^. DAMTC enabled maintenance of intact crypt-villus structures from day 3 through day 21 in TBI mice and significantly, alleviated radiation-induced structural injury in the intestine (Fig. 1). Further, DAMTC restored absorptive capacity to baseline levels in TBI mice (Fig. 2), thereby, facilitating regain of intestinal functional ability following irradiation. These findings provided preliminary evidence for the mitigation of GI-ARS by DAMTC.

Apoptotic cell death of intestinal crypt epithelial cells and villus stromal cells is an inevitable cell fate following IR-induced damage that is a major contributing factor for GI-ARS^35–37^. A profound decrease (>20 fold) in apoptotic crypt cells (beginning day 3 until day 14), followed by ~9 fold reduction at day 21 in TBI mice (Fig. 3) as well as, a decrease (20–48 fold) in the death of stromal cells in the intestinal villus suggest that reduction in IR-induced ICPS and villus stromal cell death form important aspects of mitigating radiation-induced GI-ARS by DAMTC. Decline in the levels of active caspase-3 and cPARP observed (Fig. 4) strongly suggested subjugation of intrinsic apoptotic pathway by DAMTC, which was supported by changes in the levels of Bax and Bcl-2 (Fig. 4).

Resurrection of the intestinal epithelium depends on the sustenance and regeneration of intestinal crypt stem (ICS) cells following radiation-induced intestinal cell ablation and structural disorganization^5,38–40^. Reduction in IR-induced apoptotic death of ICPS cells and rate of replenishment of the ICS cells are important factors that determine the extent of jejunal recovery following TBI^45,46^. Interestingly, DAMTC enhanced PCNA expression in TBI mice as early as 3 days post TBI (Fig. 5), suggestive of enhanced proliferation status, which appears to be a contributing factor for the re-population and regeneration of cycling cells in the jejunum (mostly the ICS). Thus, inhibition of IR-induced apoptotic death synergize with repopulation of ICPS cells by DAMTC in overcoming IR-mediated cycling (ICS) cells depletion thereby, enabling the re-construction of intestinal epithelium.. Defect in the activation of the NF-κB canonical pathway in mice (IKKβ-deficient mice) has been shown to contribute to radio-sensitivity in the small intestine and increased mortality^39,61^. Furthermore, GI-ARS is intricately linked to the balance between anti-apoptotic and pro-apoptotic signalling cascades in the ICPS cells post TBI^61^. Enhanced NF-κB expression following TBI observed here (Fig. 5) suggests that NF-κB activation by DAMTC accounts not only for prolonging cell survival, but also for diminishing IR-induced apoptosis of cells in the intestinal jejunum. These findings corroborate our observations on the abrogation of IR-induced ICPS cell apoptosis by DAMTC (Fig. 3). Cumulatively, it is evident that DAMTC enables induction of pro-survival signal transduction, in addition to augmenting ICS cell proliferation leading to intestinal rehabilitation post TBI for mitigating GI-ARS.

Facilitative chromatin remodelling is essential for the progression of DNA repair in the intact nuclei/cells^62–65^. Functioning as an additional acetyl group donor (besides acetyl CoA), thereby altering histone acetylation and from known interactions of polyphenols with the DNA^21–26^, DAMTC appears to influence conformation condensation facilitating DNA repair following TBI, which may be one of the contributing factors for the amelioration of IR-induced GI injury by DAMTC. Our recent study demonstrated the efficacy of DAMTC in reducing residual DNA damage as well as cytogenetic damage in the hematopoietic system of TBI mice^31^. Radiation-induced perturbation in the progression of cells through the cell cycle is linked to the extent of DNA damage and regulated by p53 and various check point regulators like CHKs^47–51^. Stabilization and accumulation of p53 leading to of the modifications of damage response pathways are primary contributing factors in IR-induced crypt cell death^66–68^. Lower levels of pCHK2 and p53 in the jejunum of DAMTC-TBI mice are suggestive of a lower level of residual DNA damage (Fig. 6) and the consequent damage-linked cell death. Apoptotic death of irreparably damaged ICS cells following IR exposure is implicated as the major cause for GI-ARS^46^. Therefore, reduction in the residual DNA damage (due to facilitated DNA repair) and/ or downregulation of p53 activation by DAMTC appears to be partly responsible for reduction in the death of epithelial crypt stem cells, leading to their enhanced survival. Down-regulation of TBI-induced p21 expression by DAMTC in TBI mice (Fig. 6) lends support to this, as deletion of p21 has been shown to confer protection to ICPS cells against IR-induced apoptosis^54^. Collectively, initiation and propagation of an anti-p53- and anti-p21-dependent synergistic signal by DAMTC appears to diminish the extent of damage accumulation and apoptosis of the ICPS cells post IR exposure. Further, the enhanced levels of cyclins D1 and B1 (Fig. 6) observed under these conditions may also facilitate the repopulation by facilitating progression through the cell cycle to replenish the intestinal epithelium. Further studies are warranted to unravel the precise mechanisms underlying facilitation of DNA repair by DAMTC, an important aspect of mitigation of radiation damage.

Oxidative stress plays an important role in the manifestation of radiation damage and maintenance of redox balance is essential for facilitating tissue recovery^4^. Augmentation of anti-oxidant defence by DAMTC in the intestinal epithelium was exemplified by enhanced expression of catalase and SOD-2 (Fig. 7). Further, IR-evoked oxidative conversion of polyunsaturated fatty acids to lipid peroxides such as malondialdehyde (MDA) and thiobarbituric acid reactive substances (TBARS), are responsible for the radiation-induced lipid peroxidation with TBARS/MDA level as an indicator of chronic oxidative tissue injury post TBI^69^. Abrogation of IR-induced oxidative damage to the lipid (MDA production) by DAMTC (Fig. 7) was clearly suggestive of alleviation of TBI-induced oxidative stress. Taken together, these results clearly establish the potential of DAMTC to minimize radiation-induced oxidative damage in the intestinal jejunum by up-regulation of anti-oxidant defence, which appears to be an important contributing mechanism towards mitigation of GI-ARS.

TBI-induced GI injury results in bacterial translocation into the circulation following disintegration of mucosal barrier leading to sepsis and death in an immune-compromised condition^1,4^. Macrophages are the major players that fight infection and undergo activation in response to variety of damage stimuli to enable intestinal protection^59,60^. Enhanced levels of Arg-1 (Fig. 9) induced by DAMTC in treated-TBI mice were suggestive of possible polarization of intestinal macrophages to an anti-inflammatory (M2) phenotype which plausibly promoted enhanced tissue repair and regeneration, that mitigated immune-toxicity associated with GI-ARS. Since, arginase-1 is also expressed in cells other than macrophages (although to a much lesser extent than in macrophages)^59^, our observations are only indicative at this stage. Mechanisms involved in the activation of specific macrophage subsets by DAMTC in response to TBI and the signalling events involved needs to be elucidated to unravel DAMTC-induced immune-modulation patterns post TBI.

Alterations in the histone/protein acetylation status significantly influence the epigenetic regulation of signalling pathways related to cell and tissue responses to stress including damage caused by radiation^70,71^. Elevated levels of acetylated-H3 in the intestinal jejunum cells in DAMTC-treated mice particularly following TBI (Fig. 8), suggests that hyper-acetylation of histone H3 by DAMTC in TBI mice could be one of the pivotal stimuli functioning at the apex of the sequence of events regulating cellular responses to radiation and the mitigation of GI-ARS.

A countermeasure agent that can effectively obviate radiation-induced multiple organ failure (MOF) is considered to be more suitable in mitigating acute effects of radiation and consequently in amelioration of radiation injury^72^. Mitigation of TBI-induced hematopoietic injury (H-ARS) by DAMTC reported earlier^31^ and conclusive evidences for mitigation of GI-ARS provided in the present study as well as our preliminary observations on the mitigation of TBI-induced lung injury in mice (data not shown) are strongly suggestive of DAMTC’s potential to counter MOF. Furthermore, induction of angiogenesis via nitric oxide synthase and immune system stimulation^73^ by acetylated polyphenols [viz. 7, 8-diacetoxy-4-methylcoumarin (DAMC)] additionally corroborate the compelling reasons for advancement in further development of DAMTC as a MCM for mitigation of the acute radiation syndrome (ARS).

The time at which a countermeasure agent is effective after exposure to radiation is an important aspect of consideration in the deployment of radiation mitigators. Agents effective when administered several hours after exposure and within first 24 hours are considered highly desirable due to practical considerations related to availability and delivery at a nuclear disaster site^1^. By virtue of mitigation of the clinical components of ARS as well as its potential to ameliorate MOF, DAMTC appears to possess merits requisite for a good mitigator. Nevertheless, further studies are required to investigate DAMTC-induced temporal changes in the physiology and intermediary metabolism following radiation exposure, before contemplating DAMTC as a mitigation agent in case of accidental human exposure to radiation. It also has a potential to be developed as an adjuvant to radiotherapy for protecting the normal tissues and thus, enhance the therapeutic gain. However, its effects on normal tissues during the course of focal irradiation of tumors remain to be investigated.

**Figure 10:**
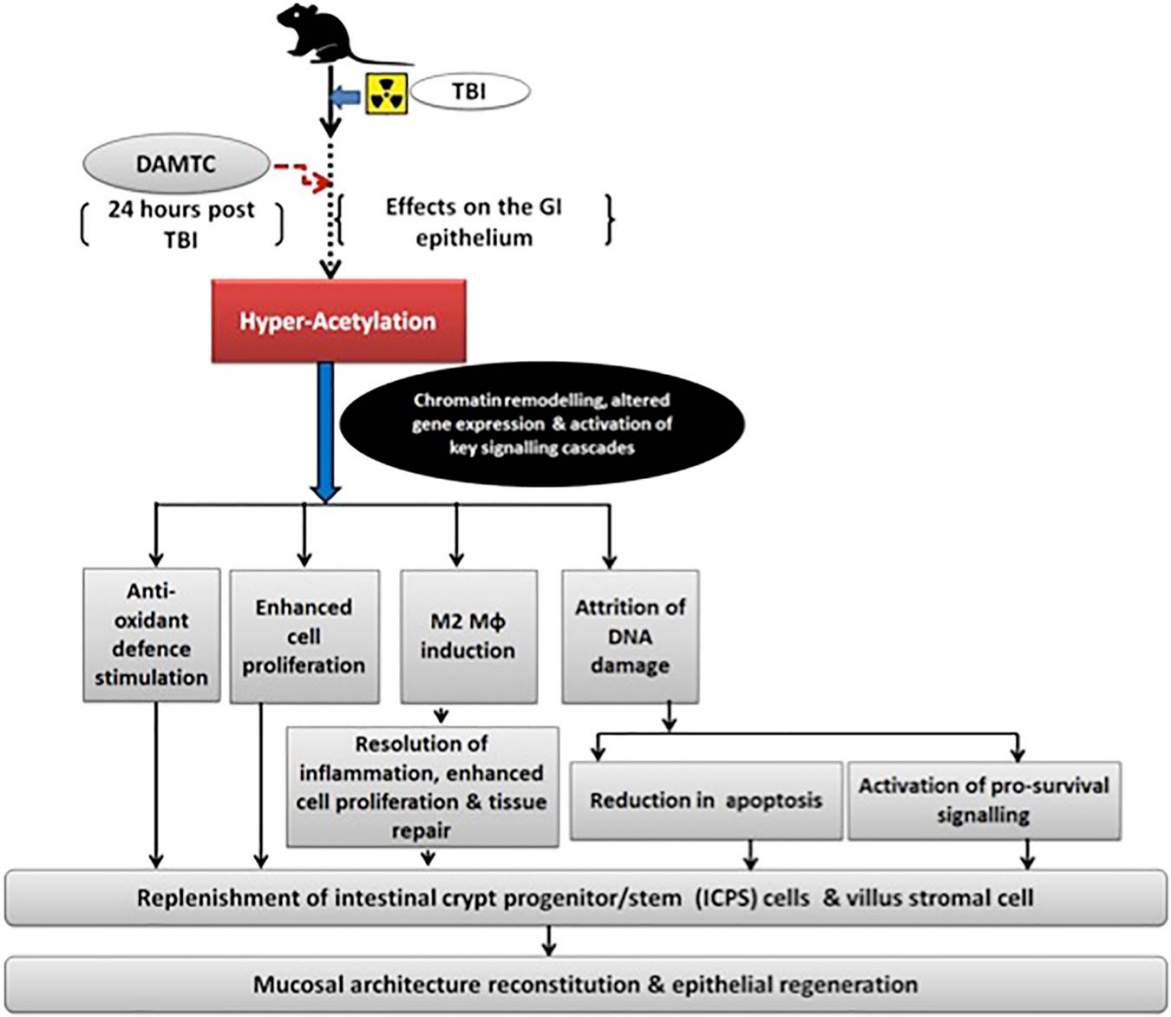
DAMTC mitigates TBI-induced gastro-intestinal injury (GI-ARS) in mice. Schematic representation of the cellular events and molecular mechanisms underlying DAMTC-mediated mitigation of GI-ARS in TBI mice.

In summary, the results of the present studies demonstrate that administration of DAMTC 24 hours post TBI effectively mitigates radiation-induced GI injury, which is contributed by multiple factors (Fig. 10); viz. (i) attenuation of radiation-induced DNA damage and apoptotic death in the intestinal epithelium, (ii) augmentation of cell proliferation and activation of pro-survival signalling in the intestinal jejunum, (iii) reduction in IR-induced oxidative damage and stimulation of anti-oxidant defence, (iv) induction of M2 macrophages in the intestinal jejunum facilitating tissue repair and regeneration, and (v) hyper-acetylation of proteins in the intestinal epithelium resulting in the replenishment of intestinal crypt progenitor/ stem (ICPS) and villus stromal cells, thereby leading to reconstitution of intestinal jejunum architecture and restoration of intestinal functionality following TBI. Mitigation of TBI-induced GI injury observed here coupled with our earlier report on the mitigation of radiation-induced hematopoietic injury suggest that DAMTC is a potential candidate molecule as countermeasure agent against acute effects of radiation, which warrants further investigations.
